# Comparative Adaptive Mechanisms of Two Indigenous Goat Genetic Resources Under Simulated Water Scarcity

**DOI:** 10.3390/ani16152432

**Published:** 2026-08-06

**Authors:** Oluwakamisi Festus Akinmoladun, Ramoello Mnyobisi, Ziyanda Mpetile

**Affiliations:** 1 Division of Agriculture and Food Technology, School of Agriculture, Geography, Environment, Ocean and Natural Sciences, The University of South Pacific, Samoa Campus, Private Bag, Apia, Samoa; 2 Department of Animal and Pasture Science, Faculty of Science and Agriculture, University of Fort Hare, Private Bag X1314, Alice 5700, Eastern Cape, South Africazmpetile@ufh.ac.za (Z.M.)

**Keywords:** water restriction, indigenous goat breeds, physiological adaptation, growth performance, serum biochemical parameters

## Abstract

Water scarcity is a major constraint to livestock production in tropical and semi-arid environments, necessitating the identification of breeds with superior adaptive capacity. This study evaluated the effects of graded water restriction (WR) on growth performance, morphometric traits, and serum biochemical responses of indigenous South African goat breeds. Thirty-six female Nguni and Xhosa goats were assigned to three water intake levels: ad libitum (W0; control), 70% (W70), and 50% (W50) of ad libitum intake in a 2 × 3 factorial arrangement. Growth performance, body measurements, and serum biochemical parameters were assessed over 75 days. Water restriction significantly reduced growth performance in both breeds (*p* < 0.05), with more pronounced effects under severe restriction (W50). However, Nguni goats maintained higher final body weight and experienced smaller reductions in average daily gain than Xhosa goats. Morphometric traits were generally less affected by WR, although Nguni goats consistently exhibited greater rump width. Biochemical responses revealed higher concentrations of sodium, chloride, alkaline phosphatase, and creatinine (*p* < 0.05) in Nguni goats, whereas Xhosa goats showed higher concentrations of urea and globulin. The findings demonstrate distinct breed-specific adaptive mechanisms to water stress, with Nguni goats exhibiting greater physiological resilience.

## Simple Summary

Water scarcity is becoming an increasingly serious challenge for livestock production, particularly in semi-arid regions where prolonged droughts and limited water availability reduce animal productivity. Indigenous goat breeds are often considered more resilient to harsh environments, but direct comparisons of their adaptive responses to water shortage remain limited. This study compared the responses of two indigenous South African goat breeds, Nguni and Xhosa, to simulated water scarcity under identical management and feeding conditions. Thirty-six growing female goats were subjected to three levels of water availability (100%, 70%, and 50% of normal intake) over a 75-day period. Growth performance, body measurements, and serum biochemical indicators were evaluated to determine breed-specific adaptation to water restriction. Water restriction reduced feed intake, body weight gain, and average daily gain in both breeds, with the greatest effects observed under severe restriction. However, Nguni goats maintained better growth performance, higher feed intake, and more stable physiological responses than Xhosa goats. While most body measurements were only minimally affected, the serum biochemical profiles indicated clear differences in how the two breeds coped with prolonged water limitation. Overall, the findings suggest that indigenous goat breeds differ in their ability to tolerate water scarcity, with Nguni goats exhibiting greater physiological resilience under the conditions of this study. These results provide valuable information for selecting and conserving resilient indigenous goat genetic resources to support sustainable and climate-resilient livestock production in water-limited environments.

## 1. Introduction

Water scarcity is one of the most pressing global challenges and is projected to intensify under future climate change scenarios [1]. Climate change poses a significant threat to the sustainability of livestock production systems due to declining water availability driven by reduced rainfall, increased temperatures, and the drying of water reservoirs, particularly in arid and semi-arid regions. These changes are expected to exacerbate existing constraints on agricultural productivity, with global food production projected to decline by approximately 10% by 2030 and over 20% by 2050 [2]. In sub-Saharan Africa, including South Africa, water scarcity is already a critical concern, driven by erratic rainfall patterns, increasing population pressure, inefficient water management, and recurrent drought events [3,4].

Water availability is a fundamental determinant of livestock productivity, particularly in small ruminant systems where it directly influences feed intake, metabolism, thermoregulation, and overall physiological function [5,6]. Water plays an essential role as a solvent in biochemical reactions, supports nutrient digestion and absorption, and is critical for maintaining heat balance. Consequently, inadequate water intake can significantly impair animal performance, growth, and health. Water scarcity, therefore, remains a major constraint to sustainable small ruminant production in water-limited environments and is expected to become increasingly severe in the future [6,7,8].

Ruminants, particularly goats, possess adaptive mechanisms that enable them to tolerate water stress more effectively than other livestock species. These adaptations include reduced water requirements associated with smaller body size, efficient rumen function that conserves water, and the ability to maintain physiological stability under dehydrated conditions [9,10]. However, the extent of this tolerance varies considerably among breeds and is largely influenced by genetic adaptation to local environmental conditions. Indigenous goat breeds, especially those originating from arid and semi-arid regions, exhibit enhanced resilience to harsh conditions, including water scarcity, due to their ability to efficiently utilise low-quality feed, travel long distances in search of resources, and maintain productivity under stress [11,12].

Among such breeds, Nguni and Xhosa goats, indigenous to the Eastern Cape of South Africa, are well adapted to semi-arid environments and represent valuable genetic resources for sustainable livestock production [9,13]. These breeds have developed complex adaptive strategies involving physiological, metabolic, and behavioural responses that enable them to cope with limited water availability. Despite their recognised adaptation to harsh environments, the mechanisms through which indigenous goat breeds respond to progressive water scarcity remain insufficiently understood. Comparative information on breed-specific adaptive strategies is limited, hindering the identification of resilient genetic resources for climate-smart livestock production in semi-arid regions.

Previous studies have investigated the effects of water restriction on individual indigenous goat breeds, including separate evaluations of Nguni and Xhosa goats under dehydration stress. However, direct comparative studies conducted under identical experimental conditions remain limited. Consequently, it remains unclear whether the physiological resilience reported for these breeds reflects inherent breed differences or variation in experimental conditions across independent studies. Furthermore, few studies have simultaneously integrated growth performance, body morphometry, and serum biochemical responses to characterise breed-specific adaptation to progressive water restriction. Addressing these gaps is essential for identifying physiological traits associated with resilience and informing breeding and management strategies for climate-resilient goat production. Therefore, the present study compared Nguni and Xhosa goats subjected to graded water restriction under identical nutritional and management conditions. By integrating performance, morphometric, and serum biochemical indicators, this study provides a comprehensive comparative assessment of breed-specific adaptive responses to progressive water scarcity.

## 2. Methods and Materials

### 2.1. Ethical Approval

The University Research and Ethics Committee of the University of Fort Hare, South Africa, approved the experimental protocol described in this study (Ref. No: MUC011SAKI01).

### 2.2. Animal Management and Experimental Design

A total of 36 clinically healthy female goats (*Capra hircus*), comprising 18 Nguni (19.43 ± 0.26 kg) and 18 Xhosa (15.92 ± 1.12 kg) breeds, were used in this study. The animals were of similar physiological status, non-pregnant, and classified as growing goats at a comparable developmental stage. All goats were sourced from the University of Fort Hare research herd in the Eastern Cape, South Africa.

Prior to the commencement of the experiment, all animals were vaccinated against foot-and-mouth disease and treated with ivermectin for parasite control. A 14-day acclimation period was observed to allow adaptation to housing, diet, and management conditions before the start of the 75-day experimental phase.

Following acclimation, the animals were randomly allocated to three water-intake treatments in a 2 × 3 factorial arrangement (breed × water level), using a simple randomisation procedure generated from a random number table. Each treatment group consisted of six goats. The treatment groups comprised ad libitum water intake (W0; control), 70% of ad libitum intake (W70), and 50% of ad libitum intake (W50). Water restriction levels were established based on the average daily water intake recorded during the acclimation period and were selected to simulate moderate and severe water stress conditions commonly encountered in semi-arid environments. Water was provided daily, and intake was determined by subtracting residual volumes from the amount offered after accounting for evaporation losses.

The experimental unit in this study was an individual animal, as goats were housed and managed individually, enabling independent measurement of feed and water intake and physiological responses. Animals were housed in well-ventilated pens (1.33 × 0.58 m), each equipped with a feeder and water trough to facilitate accurate monitoring.

Goats were offered a total mixed ration consisting of 70% lucerne hay and 30% concentrate at a feeding level equivalent to 4% of body weight on a dry matter basis. The diet was formulated to meet the nutrient requirements of growing goats according to NRC [14], and its ingredient composition and nutrient profile are presented in Table 1. Feed samples were analysed for dry matter (Method 934.01), crude protein (Method 954.01), ether extract (Method 920.39), organic matter (Method 925.05), and ash (Method 942.05) following AOAC [15] procedures. Feeding and watering were conducted at consistent times across all treatment groups.

Throughout the study, animals were monitored daily for health status and general well-being. Efforts were made to minimise pain, stress, and discomfort, including gentle handling by trained personnel and consistent management practices. Attention was given to clinical signs such as reduced feed intake, lethargy, excessive weight loss, and abnormal behaviour. Predefined humane endpoints were established, whereby animals showing severe distress, persistent anorexia, or marked physiological deterioration would be withdrawn and treated appropriately. However, no animals reached these endpoints, and no adverse events were recorded during the experimental period.

To minimise potential confounding effects, animals were maintained under uniform environmental and management conditions. Pen locations were comparable in terms of ventilation, lighting, and environmental exposure, and the order of measurements and sample collection was kept consistent across treatment groups. All procedures were conducted by the same personnel to ensure consistency and reduce experimental bias.

A formal a priori power analysis was not performed before the commencement of the study. The sample size of six animals per treatment group was determined based on animal availability, ethical considerations aimed at minimising the use of experimental animals, logistical constraints associated with intensive individual animal management, and consistency with comparable controlled studies investigating water restriction in small ruminants. Although this sample size was sufficient to detect significant differences in several performance and physiological variables, the authors acknowledge that a larger sample would improve statistical power to detect smaller treatment effects and complex interactions. This sample size provided sufficient degrees of freedom to detect significant main and interaction effects with the statistical models applied.

All selected animals met predefined inclusion criteria (clinically healthy, uniform physiological status, and within a defined weight range), which were established a priori to ensure experimental consistency. No animals or data points were excluded during the study, and all experimental units were included in the final analysis.

### 2.3. Data Collection

#### 2.3.1. Growth Measurements and Feed

Animals were fed a total mixed ration twice daily at 0900 and 1600 h, at a level equivalent to 4% of body weight (dry matter basis), to ensure consistent nutrient intake across treatments. The daily feed intake was determined by subtracting leftover feed from the total feed offered. The animals’ weights were measured weekly to monitor changes in body weight. Upon completion of the trial, total weight gain (TWG) was calculated [final weight − initial weight], while average daily gain [TWG/days of trial] was determined.

#### 2.3.2. Linear Body Morphometrics

A graduated measuring tape was used to reliably measure the body morphometrics, with values accurately recorded to the nearest centimetre. Each goat in each experimental group was measured, enabling precise evaluation and comparison of their physical characteristics across cohorts. After each animal was carefully secured, measurements were taken following the procedure outlined by Salako [16]. Measured morphometrics parameters included body length (BL: distance measured from the occipital bone and at the base of the tail), rump height (RH: the perpendicular height extending from the ground to the rump region), rump length (RL; distance between the hip point and ischium bone), rump width (RW; measured along the dorsum from one end of the lumber bone to the other), tail length (TL: the length extending from the tails base to the rear of the coccygeal bones), ear length (EL: the length from ear tip to where it attaches to the head), head width (HW: the width measured across both sides of the heads), head length (HL: distance from the area just above the horns extending from the snout or lip tip), canon bone circumference (CBC: circumference measured around the canon bone, situated between the knee and ergot), sternum height (SH: vertical distance from the ground and the underside of the sternum), withers height (WH: the vertical measurement from the highest point of the shoulders to the ground), and heart girth (HG: the circumference of the body just behind the fore leg). Triplicate measurements were performed for each dimension to enhance precision, and the mean value was subsequently recorded in centimetres for precise documentation.

#### 2.3.3. Blood Sampling and Biochemistry

Blood samples were collected from three goats per treatment per breed on days 30, 60, and 75 of the experimental periods to evaluate the effects of water restriction and breed on physiological responses. Approximately 5–10 mL of blood was obtained via jugular venipuncture using sterile needles and vacutainer tubes. Samples intended for serum biochemical analysis were collected into plain tubes, allowed to clot at room temperature, and subsequently centrifuged at 3000 rpm for 10 min to obtain serum. The harvested serum was carefully decanted into labelled microtubes and stored at −20 °C pending analysis.

Serum biochemical parameters were determined using standard colourimetric methods with commercially available diagnostic kits, following the manufacturer’s instructions. Serum biochemical parameters included electrolytes (sodium and chloride), metabolites (glucose, urea, and creatinine), proteins (total protein, albumin, and globulin), liver function indicators (bilirubin and alkaline phosphatase), and lipid profile components (cholesterol, triglycerides, HDL, and LDL). These measures were used to evaluate physiological and metabolic responses to water restriction.

To minimise analytical variation, all samples from the same animal were analysed within the same assay batch where possible. Laboratory procedures adhered to standard operating protocols for clinical biochemistry, and all measurements were performed in triplicate. Due to the nature of the treatments, blinding was not feasible during allocation, animal management, outcome assessment, or data analysis. However, objective measurement procedures and standardised protocols were applied consistently across all treatment groups to minimise potential bias.

### 2.4. Statistical Analysis

All statistical analyses were performed using IBM SPSS Statistics (v 20, IBM Corp., Armonk, NY, USA). Prior to analysis, data were assessed for normality and homogeneity of variance using the Shapiro–Wilk test and Levene’s test, respectively. In addition, residual plots were visually inspected to confirm the suitability of model assumptions. Where necessary, data transformations were considered; however, all variables met the assumptions of the applied statistical models and were analysed without transformation.

Growth performance data were analysed using a two-way analysis of covariance (ANCOVA), with breed and water restriction level as fixed factors and initial body weight included as a covariate. Morphometric traits were analysed using two-way analysis of variance (ANOVA), with breed and water restriction level as fixed effects.

Serum biochemical parameters were analysed using a linear mixed-effects model, with breed, water restriction level, time, and their interactions included as fixed effects, and animal (Goat ID) included as a random effect to account for repeated measurements within individuals. The repeated effect of time was modelled using an appropriate covariance structure, with an unstructured covariance structure initially fitted and alternative structures evaluated based on the Akaike Information Criterion (AIC). Based on the lowest Akaike Information Criterion (AIC), an autoregressive [AR (1)] covariance structure was selected for the final mixed-effects model and used for all repeated-measures analyses. Each serum biochemical parameter was analysed as a separate predefined physiological outcome using an individual mixed-effects model. Therefore, no additional experiment-wise adjustment across different biochemical variables was applied, while Bonferroni adjustment was used for pairwise comparisons of estimated marginal means within each analysis.

The ANCOVA model is expressed as follows.

*Y_ijk_* = *µ* + *A_i_* + *B_j_* + (*AB*)*_ij_* + *C_k_* + *ƹ_ijk_*

where *Y_ijk_* is the dependent variable (e.g., final weight, weight gain, etc.), µ is the overall mean, *A_i_* is the effect of the i-th level factor A (goat breed), *B_j_* is the effect of the j-th level factor B (water stress level), and *AB_ij_* is the interaction effect between breed and water stress, C_k_ is the covariate (initial weight), and *ƹijk* is the residual error term. Where significant effects were detected (*p* < 0.05), estimated marginal means were compared using the Bonferroni adjustment for multiple comparisons.

The two-way ANOVA model is

*Y_ijk_* = *µ* + *A_i_* + *B_j_* + (*AB*)*_ij_* + *ƹ_ijk_*

where *Y_ijk_* is the dependent variable (e.g., BL, HW, SH, etc.), *µ* is the overall mean, *A_i_* is the effect of the i-th level factor A (goat breed), *B_j_* is the effect of the j-th level factor B (water stress level), and *AB_ij_* is the interaction effect between breed and water stress, and *ƹ_ijk_* is the residual error term.

## 3. Results

### 3.1. Growth Performance and Linear Body Indices

Table 2 and Table 3 summarise the growth performance and linear body indices of Xhosa and Nguni goats across three levels of water stress: W0 (Control), W70, and W50. There is a significant decrease (*p* < 0.05) in final weight with increasing water stress for both breeds. Nguni goats maintained higher final body weights than Xhosa goats under W70 and W50. Similarly, the significant decrease (*p* < 0.05) in ADG due to water restriction was smaller in Nguni goats than in Xhosa goats [W70: −11.61 kg vs. −25.19 kg and W50: −13.68 kg vs. −34.75 kg]. Both total gain and ADG were significantly affected by breed, water restriction levels, and their interactions (*p* < 0.05). Nguni goats showed a slight decrease in weight gain and ADG as water restriction increased, compared with Xhosa goats. At W70, Nguni goats lost less weight (−0.87 kg) compared to Xhosa goats (−1.89 kg), and at W50, losses were −1.02 kg vs. 2.61 kg, respectively (*p* < 0.05). BW^0.75^ was also significantly affected by breed and water restriction (*p* < 0.05), with Nguni goats maintaining higher BW^0.75^ under water restriction. Both total and daily dry matter intake were significantly influenced by breed and water restriction levels *p* < 0.05). Nguni goats consistently consumed more dry matter than Xhosa goats across all water treatments.

Breed significantly (*p* < 0.05) affected tail length, rump length, width and height, body length, and sternum height. Water restriction significantly (*p* < 0.05) affected only the heart girth and body length. No significant interaction (*p* > 0.05) effect was observed for most linear body traits. Nguni goats had a wider rump width (*p* < 0.05) (average 10.67–11.00 cm) compared to Xhosa goats (8.67–9.33 cm) across all water treatments (*p* < 0.05).

Rump height and body length were higher in Nguni goats than in Xhosa at each water level (*p* < 0.05). Sternum height differed significantly between breeds (*p* < 0.05), with Nguni goats maintaining higher values. Other traits, including wither height, head width, head length, ear length, and cannon bone circumference, did not differ significantly (*p* > 0.05) by breed, water restriction, or their interaction.

### 3.2. Serum Biochemical Responses of Nguni and Xhosa Goats Under Water Stress

The effects of breed and water restriction on serum biochemical parameters of indigenous goats are presented in Table 4. Significant (*p* < 0.05) breed differences were observed in most of the biochemical indices, indicating differential physiological responses of Nguni and Xhosa goats to water stress. Nguni goats exhibited significantly higher sodium (Na) concentrations (*p* < 0.05) than Xhosa goats, particularly under severe water restriction (W50). A similar trend was observed for chloride (Cl), with Nguni goats showing significantly higher concentrations (*p* < 0.05) than Xhosa goats as water restriction increased. Creatinine levels were significantly higher (*p* < 0.05) in Nguni goats than in Xhosa goats, especially under W50. In contrast, glucose concentration did not differ significantly (*p* > 0.05) between the two breeds, although it decreased with increasing water restriction in both breeds.

Marked breed differences (*p* < 0.05) in urea concentration were observed, with Xhosa goats recording significantly higher values than Nguni goats across all water restriction levels. Total protein was also significantly (*p* < 0.05) higher in Xhosa goats under control conditions (W0), whereas both breeds exhibited increased values with increasing water stress, with Nguni goats slightly exceeding Xhosa goats under severe restriction (W50). Albumin concentration was significantly influenced by breed (*p* < 0.05), with Xhosa goats generally showing higher values than Nguni goats, although the differences were less pronounced across water treatments.

Similarly, globulin concentrations were significantly higher (*p* < 0.05) in Xhosa goats than in Nguni goats under moderate and severe water restriction. No significant (*p* > 0.05) breed differences were observed in bilirubin concentration. However, alkaline phosphatase (ALP) activity was significantly higher (*p* < 0.05) in Nguni goats than in Xhosa goats across all water restriction levels, with a pronounced increase under severe restriction. For lipid profile parameters, Nguni goats exhibited significantly (*p* < 0.05) higher cholesterol levels under control conditions. Triglyceride concentrations were significantly higher (*p* < 0.05) in Xhosa goats than in Nguni goats, particularly under control conditions, but decreased with increasing water restriction in both breeds. High-density lipoprotein (HDL) levels did not differ significantly (*p* > 0.05) between breeds, although both breeds showed an increase under moderate restriction. In contrast, low-density lipoprotein (LDL) concentrations were significantly (*p* < 0.05) higher in Nguni goats compared to Xhosa goats, especially under severe water restriction.

A significant (*p* < 0.05) three-way interaction was observed for most serum biochemical parameters, including sodium (Na), chloride (Cl), creatinine, glucose, urea, total protein, globulin, bilirubin, alkaline phosphatase (ALP), cholesterol, triglycerides, high-density lipoprotein (HDL), and low-density lipoprotein (LDL) (Table S1). However, the three-way interaction for albumin was not significant (*p* > 0.05), suggesting that albumin levels were relatively stable across breeds, water restriction levels, and sampling periods. Detailed mixed-model statistics are provided in Supplementary Table S1.

## 4. Discussion

While previous studies have investigated the effects of water restriction in individual goat breeds, the present study advances the field by directly comparing adaptive responses between Nguni and Xhosa goats across multiple physiological levels. The integrated evaluation of growth performance, body morphometry, and serum biochemical responses provides a broader understanding of breed-specific resilience mechanisms in response to progressive water scarcity. Such information is essential for identifying adaptive traits that can support climate-resilient breeding and management programmes in water-limited production environments.

Adaptive capacity reflects an organism’s ability to maintain physiological homeostasis and functional integrity under environmental stress. In water-limited environments, this capacity is critical for sustaining growth and productivity, particularly in small ruminants such as goats, which are widely recognised for their resilience to dehydration [11,12,17].

The present study demonstrated that water restriction significantly impaired growth performance in both Nguni and Xhosa goats, as evidenced by reductions in final body weight, total weight gain, and average daily gain (ADG), with the most severe effects observed under W50. These findings are consistent with previous reports indicating that dehydration disrupts feed intake, nutrient assimilation, and metabolic efficiency, ultimately leading to negative energy balance and tissue mobilisation [5,12,18,19].

The decline in growth performance under water restriction can be mechanistically linked to reduced voluntary feed intake and altered rumen function. Water plays a central role in rumen fermentation, digesta passage, and microbial activity; thus, restricted intake limits digestive efficiency and nutrient availability [20]. In addition, dehydration triggers endocrine responses, including increased secretion of antidiuretic hormone and activation of the renin–angiotensin–aldosterone system, which prioritise water conservation at the expense of growth processes [21]. These physiological adjustments reduce anabolic activity and promote catabolism, contributing to weight loss under prolonged water stress [12].

The observed reduction in total dry matter intake (TDMI) across increasing levels of water restriction also explains the decline in growth performance. Reduced feed intake under dehydration is a well-documented response, reflecting both physiological constraints and behavioural adaptations aimed at minimising metabolic heat production and water loss [19]. However, the ability of Nguni goats to maintain feed intake under water restriction suggests an adaptive strategy that supports nutrient acquisition during periods of water scarcity.

Despite the overall decline in performance, clear breed-specific differences were evident. Xhosa goats exhibited greater weight loss and more pronounced reductions in ADG under severe water restriction than Nguni goats, indicating greater sensitivity to dehydration. In contrast, Nguni goats maintained relatively higher body weights and exhibited a slower decline in growth performance, suggesting more efficient adaptive mechanisms. These may include enhanced rumen water retention, improved nitrogen recycling, and lower basal metabolic rates, traits commonly associated with indigenous breeds adapted to harsh environments [19,22].

Morphometric traits exhibited greater stability compared to growth parameters, indicating that skeletal characteristics are less sensitive to short-term environmental stress. Nevertheless, specific traits such as body length and heart girth were significantly affected by water restriction. The reduction in body length reflects suppressed growth due to reduced nutrient availability, while the more pronounced decrease in heart girth in Xhosa goats suggests greater loss of body reserves. In contrast, the relatively stable heart girth observed in Nguni goats indicates better preservation of body condition, supporting their superior adaptive capacity. These findings align with previous studies demonstrating that water deprivation negatively affects body condition and structural traits in goats, with breed-dependent variability [5,23].

Other morphometric traits, including rump width, sternum height, cannon bone circumference, and head dimensions, remained largely unaffected by water restriction, suggesting that these parameters are predominantly governed by genetic factors and are less responsive to short-term environmental stress.

The biochemical responses revealed clear breed-specific differences, reflecting distinct physiological strategies in response to dehydration stress. Nguni goats exhibited higher sodium (Na) and chloride (Cl) concentrations, particularly under severe restriction, indicating enhanced osmotic regulation and fluid conservation capacity. Elevated electrolyte levels under dehydration are primarily attributed to hemoconcentration and reduced plasma volume [24], and their higher levels in Nguni goats suggest superior regulation of fluid balance.

Serum creatinine concentrations were higher in Nguni goats, particularly under severe water restriction. This increase may reflect dehydration-associated hemoconcentration or reduced renal perfusion and clearance; however, serum creatinine is also influenced by body size and muscle mass, which differed between the breeds. In contrast, Xhosa goats exhibited higher serum urea concentrations. Although elevated urea may be associated with increased protein turnover during dehydration [25], it is also influenced by dietary crude protein concentration, feed intake, nitrogen recycling, hemoconcentration and renal handling. Given the marked decline in dry matter intake under water restriction, the elevated urea concentrations could reflect altered nitrogen metabolism rather than direct evidence of increased proteolysis alone. Total protein concentrations increased with water restriction, consistent with hemoconcentration, while albumin remained relatively stable, reflecting its conserved role in maintaining oncotic pressure [26].

Liver-related indicators showed limited disruption, with bilirubin concentrations not differing significantly between breeds. However, alkaline phosphatase (ALP) activity increased in Nguni goats, particularly under severe water restriction. Because ALP is a non-specific enzyme produced by several tissues, including the liver, bone, and intestinal mucosa, the observed increase may therefore reflect physiological responses to prolonged water restriction rather than a specific tissue adaptation or pathological process [27]. Lipid profile responses highlighted differences in energy metabolism between breeds. Variations in cholesterol and triglyceride levels suggest differential lipid mobilisation strategies, with reductions under water restriction likely linked to decreased feed intake and altered energy utilisation [28].

The significant breed × water restriction × time interactions observed for most biochemical parameters indicate that physiological responses to dehydration are dynamic and evolve over time. Changes in electrolytes, metabolites, and protein fractions across sampling periods reflect progressive adjustments aimed at maintaining homeostasis under sustained stress. Similar temporal patterns have been reported in small ruminants exposed to water scarcity [29,30,31].

The results suggest that Nguni goats exhibit stronger electrolyte regulation, whereas Xhosa goats appear to rely more heavily on protein metabolism and immune-related responses during prolonged water restriction. These contrasting physiological strategies underscore the importance of genetic adaptation in determining resilience to water stress and suggest that Nguni goats may possess superior tolerance to dehydration, making them better suited to production systems in water-limited environments.

The greater physiological resilience exhibited by the Nguni goats may reflect long-term natural and human selection under the harsher, water-limited environments of southern Africa, where recurrent seasonal droughts have favoured animals capable of maintaining homeostasis despite prolonged reductions in water availability. Such selective pressures are likely to have promoted adaptive traits, including more efficient water conservation, maintenance of feed intake under moderate water restriction, and greater tolerance of dehydration. Although the precise mechanisms underlying these breed differences were not directly investigated in the present study, the observed responses are consistent with evolutionary adaptation to contrasting environmental conditions. Similar breed-dependent variation in tolerance to water scarcity has been reported in other indigenous goat populations. For example, Silanikove [32] reported that Barmer and Black Bedouin goats survived under extremely infrequent watering schedules, with animals tolerating watering intervals of up to four days and three to six days in extensive desert grazing systems. Likewise, Hassan [33] demonstrated that Egyptian Baladi goats maintained approximately 35% of their normal feed intake during water deprivation, whereas El-Nouty et al. [34] observed that Aardi goats ceased feed consumption completely following two days of water deprivation. These findings support the view that indigenous goat breeds have evolved distinct physiological strategies for coping with water scarcity, although the magnitude and nature of these adaptations vary according to their evolutionary and production environments.

The present findings, therefore, extend existing knowledge by providing a direct comparison of two South African indigenous goat genetic resources under identical experimental conditions. The observed differences in growth performance, feed intake, morphometric traits and serum biochemical responses suggest that the adaptive responses of Nguni and Xhosa goats to prolonged water restriction are not uniform but rather reflect breed-specific physiological adjustments to dehydration. These findings reinforce the importance of considering within-species genetic variation when evaluating resilience to climate-related stressors and contribute to a broader understanding of adaptive diversity among indigenous livestock populations.

Despite the valuable insights provided, several limitations should be acknowledged. The study was conducted over a 75-day period under controlled conditions, which may not fully capture long-term adaptive responses or reflect variability in extensive production systems. Additionally, using only female goats at a specific physiological stage limits the generalisability of the findings to other sexes and production classes. Furthermore, while goats are an appropriate model for studying adaptation to water stress, caution is warranted when extrapolating these findings to other species or production systems.

## 5. Conclusions

Water restriction significantly affected growth performance, morphometric traits, and serum biochemical parameters in both Nguni and Xhosa goats, underscoring the importance of water availability in maintaining productivity and physiological function in small ruminants. Increasing levels of water restriction reduced feed intake and growth performance, with the greatest effects observed under severe restriction (W50).

Within the conditions of the present study, Nguni goats exhibited greater physiological resilience to prolonged water restriction than Xhosa goats, as evidenced by better maintenance of growth performance and serum biochemical responses consistent with more effective osmotic adjustment and metabolic homeostasis. In contrast, Xhosa goats showed greater reductions in growth performance and biochemical changes suggestive of increased protein catabolism. These findings suggest that the two indigenous South African goat breeds differ in their physiological responses to prolonged water restriction.

Although morphometric traits were generally less responsive to water restriction, reductions in body length and heart girth indicate that prolonged water limitation may influence structural development and body condition. Overall, the present findings contribute to the understanding of breed-specific responses to water scarcity and highlight the potential value of indigenous goat genetic resources for climate-resilient livestock production. Nevertheless, given the relatively small sample size, inherent breed differences, and variation in initial body weight, further studies involving larger populations and additional physiological measurements are needed to validate these findings and strengthen their application to breeding and management programmes.

## Figures and Tables

**Table 1:** Composition and nutrient content of the experimental diet (g/kg as fed).

Ingredient	Quantity
Lucerne	700
Maize gluten	166.3
Sunflower husk	127.3
MCDP	2.3
Limestone	2.1
Salt	1.5
Premix *	0.6
Calculated composition	
Organic matter	889.5
Nitrogen-free extract	440
Ether extract	17.5
Crude fibre	215.3
CP	216.7
Phosphorous	3.8
Calcium	15.7
Magnesium	6.0
Potassium	8.3
Sodium mg/kg	2263
Copper mg/kg	41
Iron mg/kg	116
Manganese mg/kg	38
Zinc mg/kg	18

MCDP = Monosodium diphosphate. * Ca—220 g/kg; P—55 g/kg; Mg—35 g/kg; S—22 g/kg; Cl—105 g/kg; Na—70 g/kg; Mn—1500 mg/kg; Fe—500 mg/kg; Zn—1550 mg/kg; Cu—440 mg/kg; Co—50 mg/kg; I—40 mg/kg; Se—20 mg/kg.

**Table 2:** Least-squares means (±SEM) of growth performance of Nguni and Xhosa goats subjected to graded water restriction.

Variables	Xhosa Goat			Nguni Goats			SEM	*p*-Value		
	W0	W70%	W50	W0	W70	W50		B	W	BxW
IW (kg) *	15.70	15.63	16.18	19.20	19.63	19.47	2.044	0.052	0.984	0.985
FW (kg)	19.68 ^a^	15.74 ^c^	15.03 ^d^	19.41 ^a^	16.76 ^b^	16.60 ^b^	0.186	0.001	0.000	0.001
Gain (kg)	2.05 ^a^	−1.89 ^c^	−2.61 ^d^	1.78 ^a^	−0.87 ^b^	−1.02 ^b^	0.186	0.001	0.000	0.001
ADG (g/d)	27.33 ^a^	−25.19 ^c^	−34.75 ^d^	23.72 ^a^	−11.61 ^b^	−13.68 ^b^	2.488	0.001	0.000	0.001
BW^0.75^ (kg^0.75^)	9.33 ^a^	7.84 ^d^	7.58 ^e^	9.22 ^b^	8.27 ^c^	8.21 ^c^	0.069	0.001	0.000	0.001
TDMI (kg)	46.39 ^b^	27.96 ^e^	24.55 ^f^	53.36 ^a^	41.75 ^c^	39.06 ^d^	0.594	0.000	0.000	0.000
TDMI/d (g/d)	618.53 ^b^	372.84 ^e^	327.29 ^f^	711.46 ^a^	556.66 ^c^	520.83 ^d^	7.923	0.000	0.000	0.000
TDMI/d/BW^0.75^ (g/kg^0.75^/day)	68.08 ^b^	49.16 ^e^	43.92 ^f^	76.79 ^a^	67.14 ^c^	63.46 ^d^	1.137	0.000	0.000	0.001

^a–f^ Means with different superscripts across the row are significantly different (*p* < 0.05); IW: initial weight; FW: final weight; ADG: average daily gain. TDMI: total dry matter intake; BW: body weight; *: the IW weight, although not significant, was used as a covariate; B: effect of breed. W: water restriction percentages.

**Table 3:** Least-squares means (±SEM) of linear body indices of Nguni and Xhosa goats subjected to graded water restriction.

Variables (cm)	Xhosa Goat			Nguni Goats			SEM	*p*-Value		
	W0	W70	W50	W0	W70	W50		B	W	BxW
Tail length	12.33 ^ab^	11.67 ^c^	11.33 ^c^	13.67 ^a^	13.33 ^ab^	13.33 ^ab^	0.850	0.033	0.838	0.231
Rump width	9.33 ^b^	9.00 ^b^	8.67 ^b^	11.00 ^a^	10.33 ^a^	10.67 ^a^	0.430	0.000	0.335	1.000
Rump length	13.00 ^bc^	12.00 ^c^	12.00 ^c^	14.33 ^a^	13.68 ^ab^	12.67 ^c^	0.624	0.033	0.267	0.338
Rump height	55.33 ^bc^	54.00 ^bc^	54.33 ^bc^	60.67 ^a^	59.67 ^ab^	59.67 ^ab^	2.389	0.016	0.871	0.997
Heart girth	72.67 ^a^	67.33 ^b^	64.67 ^c^	71.33 ^a^	69.33 ^a^	69.00 ^a^	1.284	0.138	0.005	0.105
Body length	52.00 ^bc^	51.67 ^c^	51.00 ^c^	58.33 ^a^	55.33 ^b^	53.33 ^b^	0.828	0.000	0.012	0.086
Wither height	53.68	53.33	53.00	54.33	53.33	53.33	1.627	0.806	0.865	0.979
Sternum height	28.67 ^ab^	27.00 ^b^	27.33 ^b^	32.00 ^a^	30.33 ^a^	29.33 ^ab^	1.186	0.011	0.236	0.813
Head width	10.33	10.33	9.00	9.67	9.33	9.00	0.593	0.274	0.397	0.397
Head length	17.68	17.33	16.67	18.33	18.00	18.00	0.828	0.213	0.729	0.898
Ear length	17.33	17.00	16.67	16.00	16.17	16.17	0.430	0.260	0.729	0.729
CBC	7.33	7.33	7.00	8.33	8.33	8.00	0.638	0.079	0.836	1.000

CBC: canon bone circumference; W: water restriction percentages; ^a–c^ Means with different superscripts across the row are significantly different (*p* < 0.05). B: effect of breed; W: water restriction percentages.

**Table 4:** Least-squares means (±SEM) of serum biochemical indices of Nguni and Xhosa goats subjected to graded water restriction.

Variables	Xhosa Goat			Nguni Goats			SEM	*p*-Value		
	W0	W70	W50	W0	W70	W50		B	W	BxW
Na (mmol/L)	136.22 ^b^	137.33 ^ab^	139.89 ^ab^	137.42 ^ab^	138.42 ^ab^	142.67 ^a^	0.236	<0.001	<0.001	0.030
Cl (mmol/L)	100.74 ^b^	103.33 ^b^	105.04 ^ab^	103.42 ^b^	105.00 ^ab^	108.17 ^a^	0.283	<0.001	<0.001	0.410
Creatinine (µmol/L)	45.56 ^d^	47.44 ^c^	51.22 ^b^	45.83 ^d^	49.67 ^b^	57.33 ^a^	0.533	<0.001	<0.001	<0.001
Glucose (mmol/L)	2.90	2.69	2.42	2.93	2.66	2.60	0.053	0.179	<0.001	0.146
Urea, (mmol/L)	6.93 ^c^	9.22 ^b^	10.54 ^a^	3.33 ^d^	4.27 ^d^	4.33 ^d^	0.069	<0.001	<0.001	<0.001
Total protein (g/L)	51.23 ^e^	60.96 ^c^	64.11 ^a^	58.67 ^d^	59.92 ^c^	64.67 ^a^	0.380	<0.001	<0.001	<0.001
Albumin (g/L)	13.19	13.82	14.55	12.06	12.33	14.33	0.312	0.010	<0.001	0.128
Globulin (g/L)	38.00 ^d^	47.39 ^b^	51.33 ^a^	46.38 ^b^	47.33 ^b^	49.15 ^a^	0.396	<0.00	<0.001	<0.001
Bilirubin (µmol/L)	5.77	5.96	5.33	5.39	5.28	5.50	0.203	<0.001	<0.001	0.541
ALP (U/L)	17.11 ^e^	18.81 ^e^	20.75 ^d^	23.83 ^c^	28.00 ^b^	34.00 ^a^	0.335	<0.001	<0.001	<0.001
Cholesterol (mmol/L)	1.21 ^c^	1.35 ^b^	1.48 ^a^	1.54 ^a^	1.39 ^b^	1.08 ^d^	0.027	<0.001	<0.001	<0.001
Triglyceride (mmol/L)	0.61 ^a^	0.26 ^b^	0.16 ^c^	0.21 ^b^	0.16 ^c^	0.13 ^d^	0.137	<0.001	<0.001	0.002
HDL (mmol/L)	0.79 ^c^	1.00 ^a^	0.93 ^a^	0.86 ^b^	0.96 ^a^	0.92 ^a^	0.012	0.823	<0.001	<0.001
LDL (mmol/L)	0.22 ^d^	0.31 ^c^	0.34 ^c^	0.30 ^c^	0.56 ^b^	0.75 ^a^	0.005	<0.001	<0.001	<0.001

^a–e^ Means with different superscripts across the row are significantly different (*p* < 0.05); HDL: high-density lipoprotein; LDL: low-density lipoprotein; ALP: alkaline phosphatase. B: effect of breed; W: effect of water restriction.

## Data Availability

The original contributions presented in this study are included in the article/Supplementary Materials. Further inquiries can be directed to the corresponding author.

## Supplementary Materials

The following supporting information can be downloaded at https://www.mdpi.com/article/10.3390/ani16152432/s1. Table S1: Summary of fixed effects (breed, water restriction, time and their interactions) from the repeated-measures mixed-effects analysis of serum biochemical variables.
