# Supplementary material for: Comparative Adaptive Mechanisms of Two Indigenous Goat Genetic Resources Under Simulated Water Scarcity
Source: Animals (Basel). 2026 Aug 6;16(15):2432. doi: 10.3390/ani16152432 (PMC13464023; doi:10.3390/ani16152432)
Supplement: Supplementary file 1 [file animals-16-02432-s001.zip › animals-4445541-supplementary.pdf]

Table S1. Summary of fixed effects (breed, water restriction, time and their interactions) from the repeated-measures mixed-effects analysis of serum biochemical variables.

Variables	Effects	Breed	Water	Time	B *W	B*T	W*T	B*W*T
Na (mmol/L)	df	34.44	34.44	23.24	34.44	23.23	23.23	23.23
	F-value	84.32	191.70	3.04	6.85	24.25	1.97	18.08
	P-value	<0.001	<0.001	0.067	0.003	<0.001	0.131	<0.001
Cl (mmol/L)	df	35.86	35.86	23.70	35.864	23.702	23.702	23.702
	F-value	116.09	127.92	23.96	3.495	0.284	5.402	9.249
	P-value	<0.001	<0.001	<0.001	0.041	0.755	0.003	<0.001
Creatinine (μmol/L)	df	31.35	31.35	27.23	31.35	27.22	27.23	27.23
	F-value	43.45	134.35	368.57	15.51	60.10	10.89	20.56
	P-value	<0.001	<0.001	<0.001	<0.001	<0.001	<0.001	<0.001
Glucose (mmol/L)	df	35.96	35.96	24.36	35.96	24.36	24.36	24.36
	F-value	1.88	29.62	16.73	2.03	14.66	2.49	3.12
	P-value	0.179	<0.001	<0.001	0.146	<0.001	0.069	0.033
Urea (mmol/L)	df	34.91	34.91	22.91	34.91	22.91	22.91	22.91
	F-value	11013.21	1551.70	994.26	1553.72	996.29	88.18	87.82
	P-value	<0.001	<0.001	<0.001	<0.001	<0.001	<0.001	<0.001
Total protein (g/L)	df	33.95	33.95	21.54	33.95	21.54	21.54	21.54
	F-value	55.62	310.61	63.51	70.19	92.19	33.81	43.76
	P-value	<0.001	<0.001	<0.001	<0.001	<0.001	<0.001	<0.001
Albumin (g/L)	df	34.38	34.38	21.80	34.39	21.80	21.80	21.80
	F-value	13.76	18.54	18.99	2.183	12.27	0.51	2.12
	P-value	0.001	<0.001	<0.001	0.128	<0.001	0.729	0.111
Globulin (g/L)	df	34.97	34.97	24.67	34.97	24.67	24.67	24.67
	F-value	41.28	211.78	32.46	98.80	9.15	17.13	21.88
	P-value	<0.001	<0.001	<0.001	<0.001	<0.001	<0.001	<0.001
Bilirubin (μmol/L)	df	30.16	30.16	26.21	30.16	26.21	26.21	26.21
	F-value	97.67	23.89	13.09	0.62	1.91	1.71	9.79
	P-value	<0.001	<0.001	<0.001	0.540	0.160	0.177	<0.001
ALP (U/L)	df	34.02	34.02	25.59	34.02	25.59	25.59	25.59

	F-value	1260.44	213.68	96.22	48.27	12.07	19.42	19.84
	P-value	<0.001	<0.001	<0.001	<0.001	<0.001	<0.001	<0.001
Cholesterol (mmol/L)	df	34.24	34.24	24.74	34.24	24.74	24.74	24.74
	F-value	13.19	34.56	339.62	153.23	93.74	7.60	21.08
	P-value	0.001	<0.001	<0.001	<0.00	<0.001	<0.001	<0.001
Triglyceride (mmol/L)	df	13.28	13.28	16.03	13.28	16.03	16.03	16.03
	F-value	26.24	20.98	90.86	10.67	106.68	120.37	93.59
	P-value	<0.001	<0.001	<0.001	0.002	<0.001	<0.001	<0.001
HDL (mmol/L)	df	31.47	31.47	27.46	31.47	27.47	27.47	27.46
	F-value	0.051	81.61	74.64	11.35	15.32	7.51	6.81
	P-value	0.823	<0.001	<0.001	<0.001	<0.001	<0.001	0.001
LDL (mmol/L)	df	35.99	35.99	24.06	35.99	24.06	24.06	24.06
	F-value	3310.56	1498.33	763.88	480.22	696.19	61.16	84.84
	P-value	<0.001	<0.001	<0.001	<0.001	<0.001	<0.001	<0.001
